# Erysipelas

**Published:** 1844-07

**Authors:** 


*Erysipelas.—Medical Society of London.*—Dr. Clutterbuck in allusion to the discussion of the last meeting, explained that he confined his remarks to the more ordinary form of erysipelas, affecting the head and face. He proceeded to remark that he considered the term erysipelas should be confined to cases in which the skin only was affected, and considered the definition of Cullen respecting the disease to be the most correct, "an inflammation of the skin tending to spread around."

We regret that we have not space to give in full the observations made by Dr. Clutterbuck, and that we must content ourselves with a summary of the copious report sent to us. The chief points insisted on were the facts that erysipelas was of the nature of all other inflammations, but modified by the structure affected—that the skin being an organ of sense influenced more or less the brain, and particularly so when the erysipelas affected the head and face. This injurious influence might amount to mere irritation, or extend to inflammation, but, generally speaking, it did not require very active treatment either by blood-letting or stimulants. He was still of opinion that the passive or palliative treatment, such as he had advocated at the last meeting, was the most likely to be generally successful.

In the conversation which followed, the subject of the contagious nature of erysipelas was discussed. No facts, however, on either side of the question were advanced, but the president was of opinion, from his own experience, that the disease never was contagious, although it might certainly become epidemic in hospitals, from persons breathing constantly the impure atmosphere surrounding those persons affected with the disease. He mentioned, also, in regard to the treatment of the affection, that a physician of much practice in London was so confident of the value of the nitrate of silver in this affection that he went so far as to assert, that when a strong solution of this chemical was applied sufficiently early over the inflamed surface it invariably succeeded in effecting a cure.—*London Lancet.*

